# Voltammetric and impedimetric determinations of selenium(iv) by an innovative gold-free poly(1-aminoanthraquinone)/multiwall carbon nanotube-modified carbon paste electrode†

**DOI:** 10.1039/d1ra07588h

**Published:** 2022-02-10

**Authors:** Asmaa Galal Ali, Mahmoud Fatehy Altahan, Amr Mohamed Beltagi, Abla Ahmed Hathoot, Magdi Abdel-Azzem

**Affiliations:** Electrochemistry Laboratory, Chemistry Department, Faculty of Science, Menoufia University Shibin El-Kom 32511 Egypt asmaa.galal081986@gmail.com; Central Laboratory for Environmental Quality Monitoring, National Water Research Center El-Qanater El-Khairia 13621 Egypt mahmoud_abdalqader@nwrc.gov.eg; Chemistry Department, Faculty of Science, Kafrelsheikh University Kafr El-Sheikh 33516 Egypt

## Abstract

Selenite (Se^4+^), a significant source of water pollution above the permissible limits, is considered a valuable metal by environmentalists. In this study, we described a novel electrochemical sensor that utilized a carbon paste electrode (CPE) that was modified using multiwall carbon nanotubes (MWCNTs) and poly(1-aminoanthraquinone) (p-AAQ) for finding Se^4+^ in water samples. Electrochemical quantification of Se^4+^ depends on the formation of a selective complex (piaselenol) with p-AAQ. In this work, we prepared a CPE modified by physical embedding of MWCNTs and 1-aminoanthraquione (AAQ), while the polymer film was formed by anodic polymerization of AAQ by applying a constant potential of 0.75 V in 0.1 M HCl for 20 s followed by cyclic voltammetry (CV) from −0.2 to 1.4 V for 20 cycles. The modified CPE was used for differential pulse voltammetry (DPV) of Se^4+^ in 0.1 M H_2_SO_4_ from 0 to 0.4 V with a characteristic peak at 0.27 V. Further, the proposed sensor was characterized by scanning electron microscopy, energy-dispersive X-ray spectroscopy, and electrochemical impedance spectroscopy (EIS). The analytical conditions regarding the electrode performance and voltammetric measurements were optimized, with the accumulation time and potential, supporting electrolyte, differential-pulse period/time, and amplitude. The EIS results indicated that the p-AAQ/MWCNTs-modified CPE sensor (p-AAQ/MWCNTs/CPE) that also exhibited low charge-transfer resistance (*R*_ct_) toward the anodic stripping of Se^4+^, exhibited good analytical performance toward different concentrations of Se^4+^ in a linear range of 5–50 μg L^−1^ Se^4+^ with a limit of determination (LOD) of 1.5 μg L^−1^ (3*σ*). Furthermore, differential-pulse voltammetry was employed to determine different concentrations of Se^4+^ in a linear range of 1–50 μg L^−1^ Se^4+^, and an LOD value of 0.289 μg L^−1^ was obtained. The proposed sensor demonstrated good precision (relative standard deviation = 4.02%) at a Se^4+^ concentration of 5 μg L^−1^. Moreover, the proposed sensor was applied to analyze Se^4+^ in wastewater samples that were spiked with Se, and it achieved good recovery values.

## Abstract 2

The selenite ion is quantified electrochemically by selective complexation with poly(1-aminoanthraquione) to give a piaselenol complex on a modified p-AAQ/MWCNTs/CPE sensor.

## Graphical abstract

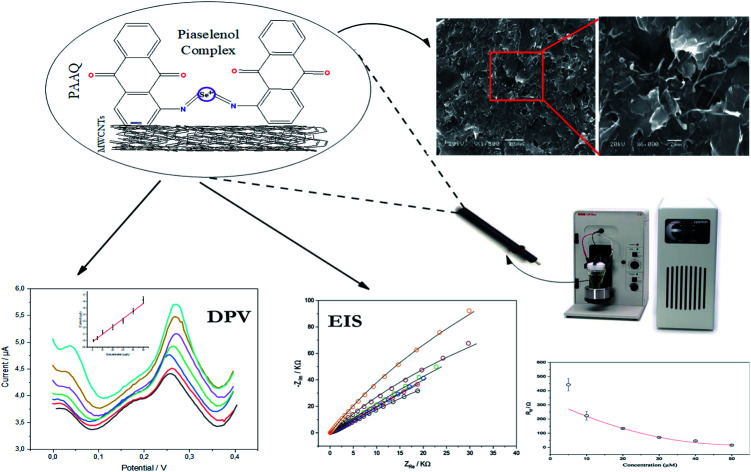

## 1. Introduction

Nowadays, environmentalists have developed a tremendous interest in the dual role of Se (a vital and simultaneously toxic element depending on its concentration in the environment and different organisms). The toxicity of Se depends mainly on its chemical form. For example, elemental Se^0^ exerts only minimum toxicological consequences on most organisms. Both selenite (Se^4+^) and selenate (Se^6+^), inorganic water-soluble species, are highly toxic to nonaccumulators because they can be absorbed by plants. Conversely, the organic forms of Se are less-toxic than the inorganic ones. Notwithstanding, Se functions as a vital element in low concentrations; it is pivotal to normal growth and development processes and functions as a defense agent against infections and cancerous diseases.^1^

The extreme exposure of living organisms to Se can cause many health challenges, such as nerve damage, chronic diseases (selenosis), gastrointestinal upsets, and white blotchy nails,^2,3^ whereas its deficiency can cause cancer, heart diseases, and muscular dystrophy.^4^ Furthermore, dissolved Se species exhibit higher mobility and bioavailability in seawater compared with the undissolved ones and can readily harm organisms and marine ecosystems.^5^ Thus, it is imperative to monitor and control the limit of Se in drinking water; the World Health Organization (WHO) has set the maximum allowable concentration of Se in drinking water as 10 μg L^−1^.^4,6^ Therefore, the sensitive, selective, and on-site determination of inorganic Se in drinking water is greatly beneficial to apply careful measures for risk management through the process of treating or controlling the anthropogenic activities of water, which introduces high concentrations of inorganic Se into an aquatic environment. There are many analytical techniques in the literature for detecting Se^4+^ at trace levels. Among them, atomic fluorescence spectroscopy,^7^ gas chromatography,^8^ high-performance liquid chromatography (HPLC),^9^ inductively coupled plasma-mass spectroscopy,^10^ neutron activation analysis,^11^ atomic absorption spectroscopy,^12^ ion chromatography,^13^ and capillary electrophoresis^14^ have been reported for the quantification of Se. Based on the significance of the in-field monitoring and *in situ* survey of Se^4+^ in water resources, previous lab-based techniques have featured limitations such as expensiveness, tedious sample preparation, and prolonged procedures. Conversely, electroanalytical techniques feature several advantages such as facile operation, inexpensiveness, rapid process, low requirement for reagents, high sensitivity, and readily moveable instruments over other analytical techniques, and these advantages form the basis for constructing portable *in situ* devices.^15,16^ Stripping voltammetry is a highly sensitive electrochemical technique for analyzing trace metals. It is based mainly on the accumulation of the metal ions on the surface of a working electrode, followed by doffing from the surface by an oxidation-assessed technique, *e.g.*, square wave voltammetry or differential-pulse voltammetry (DPV). For the electrochemical determination of Se^4+^ in aqueous environmental samples, the performance of the technique (sensitivity and selectivity) depends on the sensor material.

Over the last three decades, a variety of electrodes such as hanging mercury drop electrode (HDME)^17,18^ and solid electrodes (gold,^19^ platinum,^20^ and silver^21^) have been reported for the quantification of Se in water.

Despite the sensitivity of HDME toward Se (limit of determination (LOD) = 0.08 μg L^−1^), the high toxicity of Hg-based electrodes has limited their wide commercial application (Hg is carcinogenic). Gold electrodes also demonstrate great sensitivity toward determining Se^4+^ by forming an Ag–Se intermetallic bond in the deposition step, and this ensures the high determination of Se (an LOD value of up to 0.05 ppb^22^). However, gold-based electrodes also exhibit some drawbacks, including the surface fouling one. Thus, it requires the continuous cleaning of the surface of the electrode. Further, the features of Pt and Ag are the same as that of Au, although the utilization of those electrodes is further limited by their high cost and intensive cleaning with utmost caution. Therefore, other inexpensive alternatives are desired. Thus, carbon-based electrodes, such as a graphite electrode^23^ and a graphite screen-printed electrode can be applied for the determination of Se.^24^

In the last five decades, carbon paste electrodes (CPEs) have been widely applied to the fields of electroanalysis and biosensing because of their remarkable advantages such as low cost, facile preparation, and renewability.^25^ Further, CPEs can be modified using carbon nanotubes (CNTs) or multiwall CNTs (MWCNTs) that offer a large effective surface area and high mass transfer and catalysis. CNTs have attracted great interest because their unique properties, which combine in their high surface area ratio, nanometer dimensions, corrosion resistance, rapid electron transfer and abundance of electroactive sites, make CNTs an excellent electrode material. Previous studies have shown that CNT-based electrodes are able to reduce overvoltage and enable rapid electron transfer. Compared to bare electrode. CNT-based electrodes showed higher peak current sensitivity and selectivity towards the analytes.^26^

MWCNTs have attracted enormous interest owing to their flexibility, good mechanical strength, chemical stability, and unique electrochemical properties and their ability to improve electron-transfer reactions when they are utilized as electrode resources.^27^ Additionally, these carbon nanomaterials can enhance the sensitivity and selectivity of heavy metal ion sensors since they offer high π-conjunction and doping in organic molecules.^28^ Conducting polymers have attracted great attention owing to their unique properties and potential applications in many devices.^29,30^ Furthermore, a conducting polymer possessing functional groups that can interact with definite elements is valuable in the analytical processes. Se^4+^ demonstrates a high affinity to undergo a definite reaction with aromatic *o*-diamines to form consistent piaselenol derivatives. This reaction is the basis for the specific determination of trace Se.^31,32^ Our group has been involved in a project on the preparation of different aniline derivatives-modified electrodes and investigation of their applications in the determination of heavy metals.^33–36^

In this study, we prepared poly(1-aminoanthraquinone) (p-AAQ) on the surface of CPE that was modified using MWCNTs (p-AAQ/MWCNTs/CPE) by anodic polymerization employing an applied potential and cyclic voltammetry (CV). This is the first report on p-AAQ/MWCNTs/CPE as an efficient modified electrode for differential-pulse stripping voltammetry (DPSV) and electrochemical impedance spectroscopy (EIS) of Se^4+^ in water. P-AAQ/MWCNTs/CPE is very easy to use and cost-effective. Further, it demonstrated great sensitivity and selectivity toward the quantification of Se^4+^ by forming the characteristic piaselenol complex.

## 2. Experimental procedure

### 2.1. Apparatus

A potentiostat (Epsilon; BASi, USA) was used for CV and DPV. A conventional electrochemical cell with three electrodes, namely, the working electrode (CPE [BASi, USA]), the auxiliary/counter electrode (a 7.5 cm long Pt wire, 0.5 mm diameter) (BASi, USA), and the reference electrode [Ag/AgCl stained with 3 M potassium chloride (Ag/AgCl, 3 M KCl) (BASi, USA)]. Subsequent potentials were set according to the reference electrode. EIS measurements were performed using a model VersaSTAT 4 potentiostat (Princeton Applied Research, Oak Ridge, TN, USA) and data were processed using EC-Lab software (Bio-Logic, USA), which was also used to fit the experimental data to the equivalent circuit. Surface morphology analysis and chemical microanalysis (EDX) were performed using a scanning electron microscope (SEM) (Model: JSM-6360LA, Joel, Japan) in conjunction with energy dispersive X-ray spectroscopy (EDX).

### 2.2. Reagents and calibration standards

All reagents and calibration standards were prepared using deionized water (18.2 MΩ at 25 °C, Barnstead Smart2Pure, Thermo Scientific, USA) and analytical reagents. All glassware was thoroughly cleaned before use; it was rinsed with 0.5 M hydrochloric acid (HCl) (37%, reagent grade, Merck, USA) and then rinsed again with deionized water. All calibration standards for Se^4+^ were prepared from selenium dioxide (SeO_2_) [reagent grade, 98%, Merck, USA]. Standard solutions for Na^+^, Ca^2+^, Mg^2+^, Al^3+^, Cd^2+^, Pb^2+^, Cl^−^, SO_4_^2−^, CO_3_^2−^, and NO_3_^−^ were prepared from sodium chloride (NaCl, 99%, Merck, USA), (CaCl_2_, 98%, Merck, USA), (MgSO_4_, 98%, Merck, USA), (AlCl_3_, reagent grade, Merck, USA), (CdCl_2_·3/2H_2_O, 99%, Merck, USA), (Pb(NO_3_)_2_, 99%, Merck, USA) and (Na_2_CO_3_, 99%, Merck, USA).

### 2.3. Preparation of the modified electrodes

The preparation of the modified electrodes of PAAQ/MWCNT/CPE was carried out in several steps. First, 0.04 g of 1-aminoanthraquinone (1-AAQ) [C_14_H_9_NO_2_, 97%, Sigma-Aldrich, USA] was dissolved in a small amount of acetonitrile (CH_3_CN, Merck, USA) and then mixed with 0.56 g of graphite (particle size < 20 μm, Sigma-Aldrich, USA), 0.1 g MWCNTs (>90% carbon base, *D* × *L* = 110–170 nm × 5–9 μm) (Sigma-Aldrich, USA) and 0.3 g paraffin oil (Sigma-Aldrich, USA) in a mortar. Mixing was continued with the help of pastel for about 10 minutes to form a uniform paste. The mixture obtained was pressed into the hole at the end of an electrode holder (BASi, USA). Subsequently, the excess electrode material was removed by polishing on a piece of filter paper (Whatman, England). The resulting modified CPE was then rinsed with deionized water and placed in an electrochemical cell with reference electrode and auxiliary electrode in 0.1 M HCl (Merck, USA). Then a fixed potential of 0.75 V was applied for 20 s with stirring at 400 rpm, followed by CV into the same solution from −0.2 to 1.4 V at a scan rate of 0.1 V s^−1^ for 10 scan cycles. The resulting PAAQ/MWCNT/CPE was rinsed with deionized water and was ready to use. The PAAQ/CPE was prepared by the same procedure in which 0.04 g of 1-AAQ was dissolved in acetonitrile and mixed with 0.66 g of graphite and 0.3 g of paraffin oil.

CPE and MWCNTs/CPE were prepared by physically embedding the following components only: 0.7 g graphite and 0.3 g paraffin oil and 0.6 g graphite, 0.1 g MWCNTs and 0.3 g paraffin oil, respectively. The resulting electrodes were then rinsed with deionized water and were ready for use without further modification.

### 2.4. Electrochemical measurement of Se^4+^

Se^4+^ was determined on the modified electrodes by anodic stripping voltammetry with differential pulses in 0.1 M H_2_SO_4_ for 60 s at a deposition potential of −0.8 V. Then, the potential was scanned by DPV anodically from −0.1 to 0.5 V at a differential pulse time of 200 ms, a differential pulse amplitude of 90 mV, a pulse width of 50 ms, and a step potential of 1 mV.

EIS was performed at 10 000 to 0.1 Hz in a solution of 0.1 M KCl containing 0.1 M Fe_3_(CN)_6_^3−/4−^ and Se^4+^. Accumulation occurred at a deposition potential of −0.8 V and a deposition time of 2 min.

Limit of detection (LOD) was determined as three time the standard deviation (*σ*) of 10 lowest concentration measurement.^37^

## 3. Results and discussion

### 3.1. Electropolymerization of AAQ

**Fig. 1:**
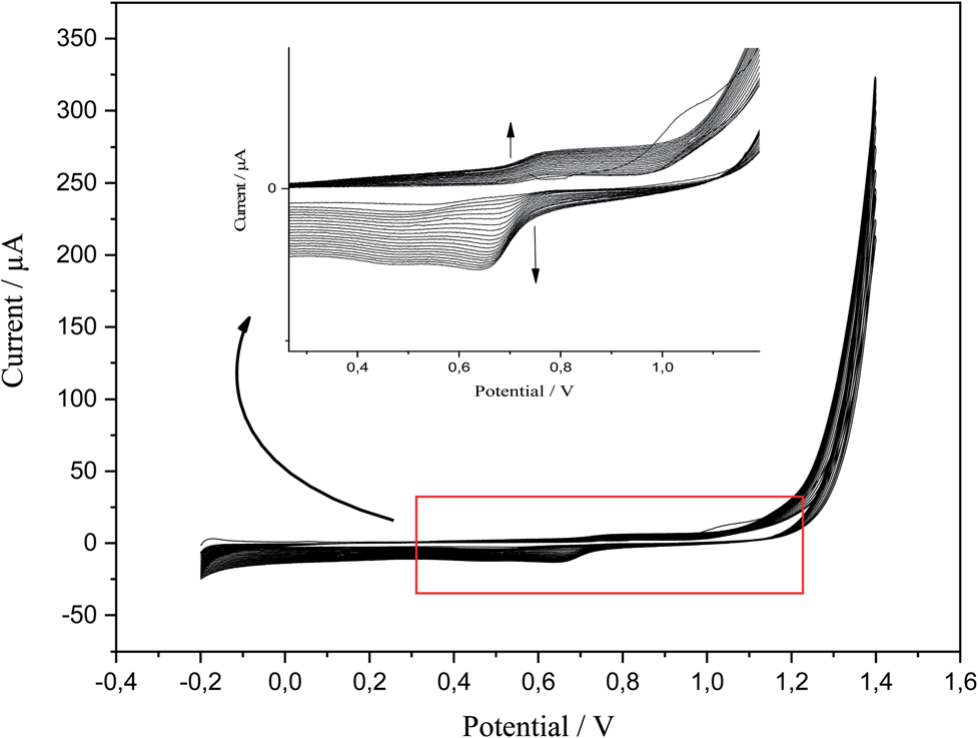
CV of p-AAQ/CPE in 0.1 M HCl at a scan rate of 0.1 V s^−1^ for 20 scanning cycles.

P-AAQ was prepared on the surface of CPE by CV; the value of the applied potential for the desired time could be determined from the first scan of CV in the electropolymerization process. According to the literature, a value of ∼0.8 V was suitable for the electrochemical preparation of the AAQ isomers.^38,39^ Anodic polymerization was performed at an applied potential of 0.75 for 20 s, followed by the activation in 0.1 M HCl *via* CV from −0.2 to 1.4 V (Fig. 1) for 20 cycles. The magnified image shows the anodic peak at 0.8 V and the cathodic one at 0.7 V during the reverse scan. During the follow-up scanning process, the two peaks increased, and this confirmed the successful formation and aggregation of the monomer molecules.

### 3.2. Differential pulse voltammetry of Se^4+^

**Scheme 1:**
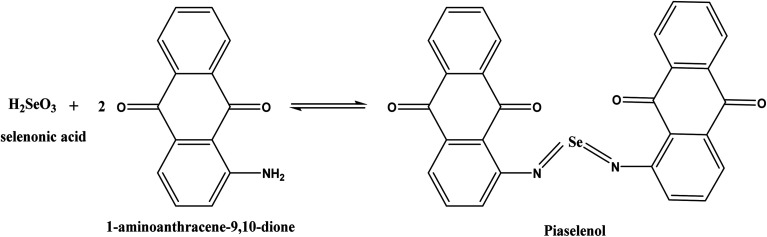
Interaction between Se and 1-AAQ to form a piaselenol complex in the preconcentration step.

The p-AAQ-modified electrodes exhibited a great affinity for Se^4+^ through the formation of a selective complex (piaselenol), which exhibited a self-accumulation tendency, as shown in Scheme 1 (ref. 1) during the preconcentration step. Thereafter, this complex was oxidized by cathodically scanning the potential and applying the differential-pulse wave-form, yielding Se^4+^ again (the stripping step).

**Fig. 2:**
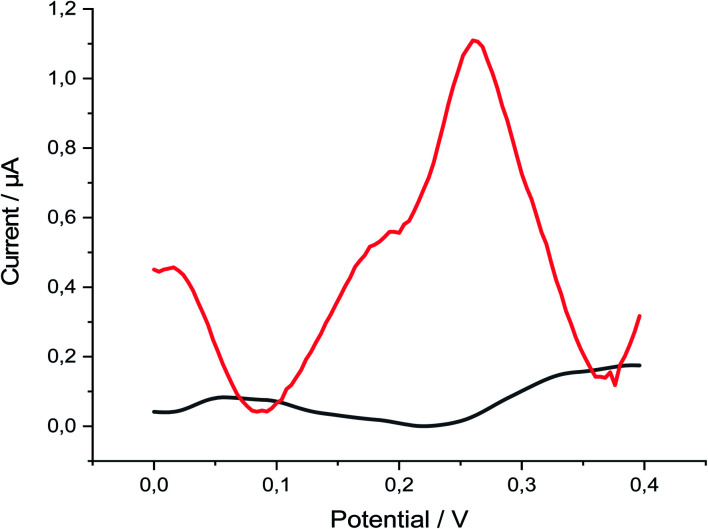
DPV of p-AAQ/MWCNTs/CPE in 0.1 M H_2_SO_4_ in the absence (black line) and presence of 30 μg L^−1^ Se^4+^ (red line), pulse time 200 ms, pulse width 50 ms, pulse amplitude 90 mV and step potential 1 mV.

P-AAQ/MWCNTs/CPE exhibited great performance toward Se^4+^ in 0.1 M H_2_SO_4_, as observed by comparing the absence and presence of 30 μg L^−1^ Se^4+^ (Fig. 2), whereas the proposed modified electrode showed three oxidation peaks at 0.02, 0.19 and 0.27 V in the differential pulse voltammogram, probably due to the oxidation of the bulk Se(0) previously deposited during the deposition step at 0.02 V, followed by a peak at 0.19 V, which is more likely due to the Se adsorbed in a monolayer by underpotential deposition (UPD),^40^ followed by the higher oxidation peak at 0.27 V, which is likely due to the selenium–nitrogen double bonds Se(N)_2_. The following equations were simplified these assumptions (eqn (1)–(3)). The highest peak current we hypothesize due to the Se(N)_2_ bonds supports our assumption that the piaselenol complex plays a key role in the analytical performance of our proposed sensor.

1 Se^0^ → Se^2+^ + 2e^−^, 0.02 V

2 Se^2+^ → Se^2+^–monolayer, 0.19 V

3 –N≔Se^2+^≕N–(piaselenol complex) → Se^6+^ + p-1-AAQ + 4e^−^, 0.27 V

**Fig. 3:**
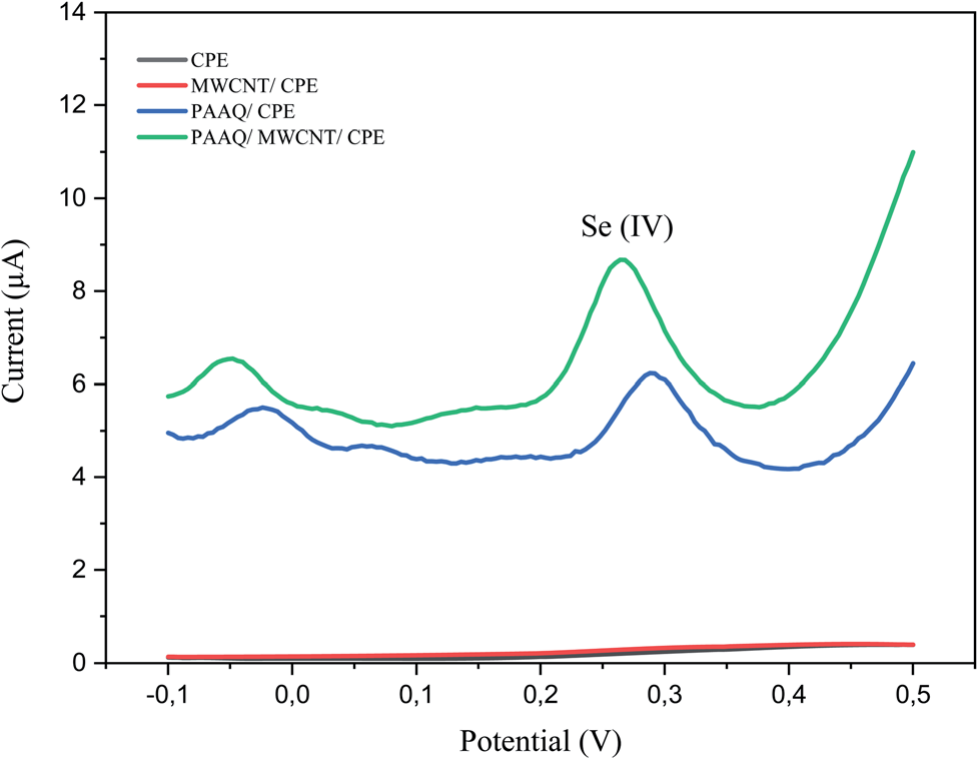
DPV of 10 μg L^−1^ Se^4+^ in 0.1 M H_2_SO_4_ on CPE, MWCNT/CPE, p-AAQ/CPE, and p-AAQ/MWCNTs/CPE, pulse time 100 ms, pulse width 50 ms, pulse amplitude 50 mV and step potential 1 mV.

A comparison of the four electrodes (CPE, MWCNTs/CPE, p-AAQ/CPE, and p-AAQ/MWCNTs/CPE) reveals the role of p-AAQ in the formation of the piaselenol compound and the determination of Se, wherein Se exhibited a different potential value (0.27 V for p-AAQ/MWCNTs/CPE) from those reported in the literature, wherein the oxidation potential of Se is in the range of 0.7–0.9 V.^4,5,41^Fig. 3 presented that Se could be detected only if the modified electrodes containing p-AAQ, CPE, and MWCNTs/CPE in the potential range of −0.1 to 0.5 did not present any peak for the solution of 0.1 M H_2_SO_4_ containing 10 μg L^−1^ Se^4+^. Moreover, the peak potential that corresponded to the oxidation of the piaselenol complex on p-AAQ/MWCNTs/CPE (0.27 V) was less positive than that on p-AAQ/CPE (0.30 V), indicating that the oxidation of the piaselenol complex was easier on the p-AAQ/MWCNTs/CPE modified electrode. Three oxidation peaks observed for p-AAQ/MWCNTs/CPE at −0.05, 0.06, and 0.27 V are likely due to the same assumption we made for the peaks in Fig. 2, with a less positive potential for the peak at 0.06, which is likely related to how fast the scanning was performed, as different DPV parameters were used for each voltammogram.

### 3.3. Characterization of modified the electrodes

**Fig. 4:**
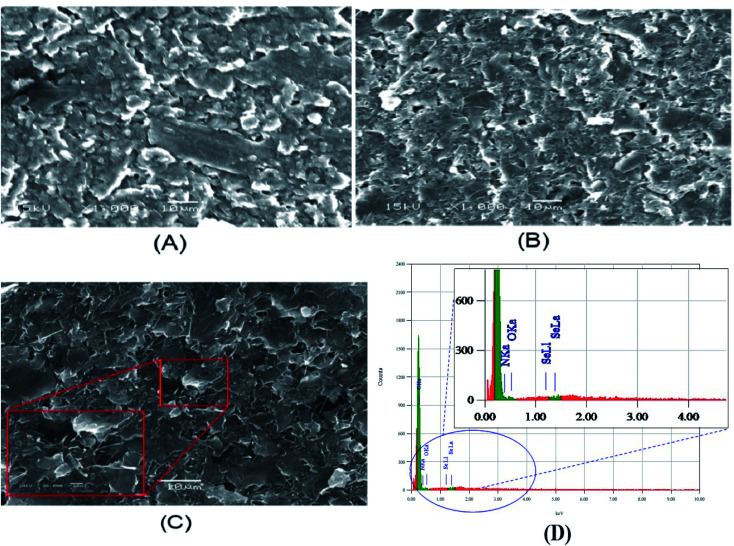
SEM images of (A) p-AAQ/CPE, (B) p-AAQ/MWCNT/CPE and (C) p-AAQ/MWCNT/CPE after the deposition of 50 μg L^−1^ Se^4+^ for 60 s at −0.8 V, and (D) EDS of p-AAQ/MWCNT/CPE after the deposition of 50 μg L^−1^ Se^4+^.

The modified electrodes (p-AAQ/CPE and p-AAQ/MWCNTs/CPE) were examined by scanning electron microscopy (SEM). The SEM enabled us to qualitatively determine the porosity and roughness of the composite surface. The images obtained (Fig. 4A and B) show the agglomerated flakes confirming the formation of a polymer network on the CPE, while the tightly intertwined MWCNTs increased the surface area, increasing the conductivity and availability of the active sites, as well as the amount of Se^4+^ that could be captured during the enrichment, improving the sensitivity. By comparing both images, p-AAQ/MWCNTs/CPE showed more rougher and less compact structure than p-AAQ/CPE. This is of course more related to the presence of MWCNTs.

In contrast, the image of p-AAQ/MWCNTs/CPE taken after the deposition of Se (Fig. 4C) shows the presence of Se by the white spot that appeared on the surface. Energy dispersive X-ray analysis (EDX) is a microanalytical elemental analysis technique associated with electron microscopy. EDX is based on the generation of X-ray patterns that show the presence of the element on the samples.

EDS clearly revealed the presence of Se, which accounted for 0.86% of the total mass, while the presence of C,N,O with a percentage of 40.1%, 55.9% and 6% showed the incorporation of Se into a piaselenol complex (Fig. 4D). The results obtained indicate successful chelation between selenide and p-AAQ.

The electrochemical behaviors of CPE, MWCNT/CPE, p-AAQ/CPE, and p-AAQ/MWCNT/CPE were tested in a 0.1 M KCl solution containing 1 mM potassium ferrocyanide. Fig. S1† shows CVs of the different electrodes in the potential range of −0.6 to 0.8 V; moreover, the active surface areas of the electrodes were calculated using the Randles–Sevcik equation as follows:

4 *I*_p_ = (2.69 × 10^5^)*n*^3/2^*AD*^1/2^*C* × *ν*^1/2^,

where *n* is the number of transferred electrons during the redox reaction, *A* is the active surface area in cm^2^, *D* is the diffusion coefficient of the molecules in the solution in cm^2^ s^−1^ (for the solution of K_4_[Fe(CN)_6_], *D* equals 6.7 × 10^−6^ cm^2^ s^−1^), and *C* is the concentration of the probe in mol cm^−3^ (1 mM). The peak current (*I*_p_), in μA, was measured at different scan rates (*ν*). Based on the Randles–Sevcik equation, the effective surface areas for the four electrodes were calculated from the slope of the linear fit between *I*_p_ and the square root of *ν*(*I*_p_/*ν*^1/2^) (Fig. S2†). Further, the calculated effective surface areas of CPE, MWCNTs/CPE, p-AAQ/CPE, and p-AAQ/MWCNTs/CPE were 0.517, 1.351, 2.726, and 4.095 cm^2^, respectively. The results indicated the enlargement of the surface area *via* incorporations of p-AAQ and MWCNTs in CPE.

**Fig. 5:**
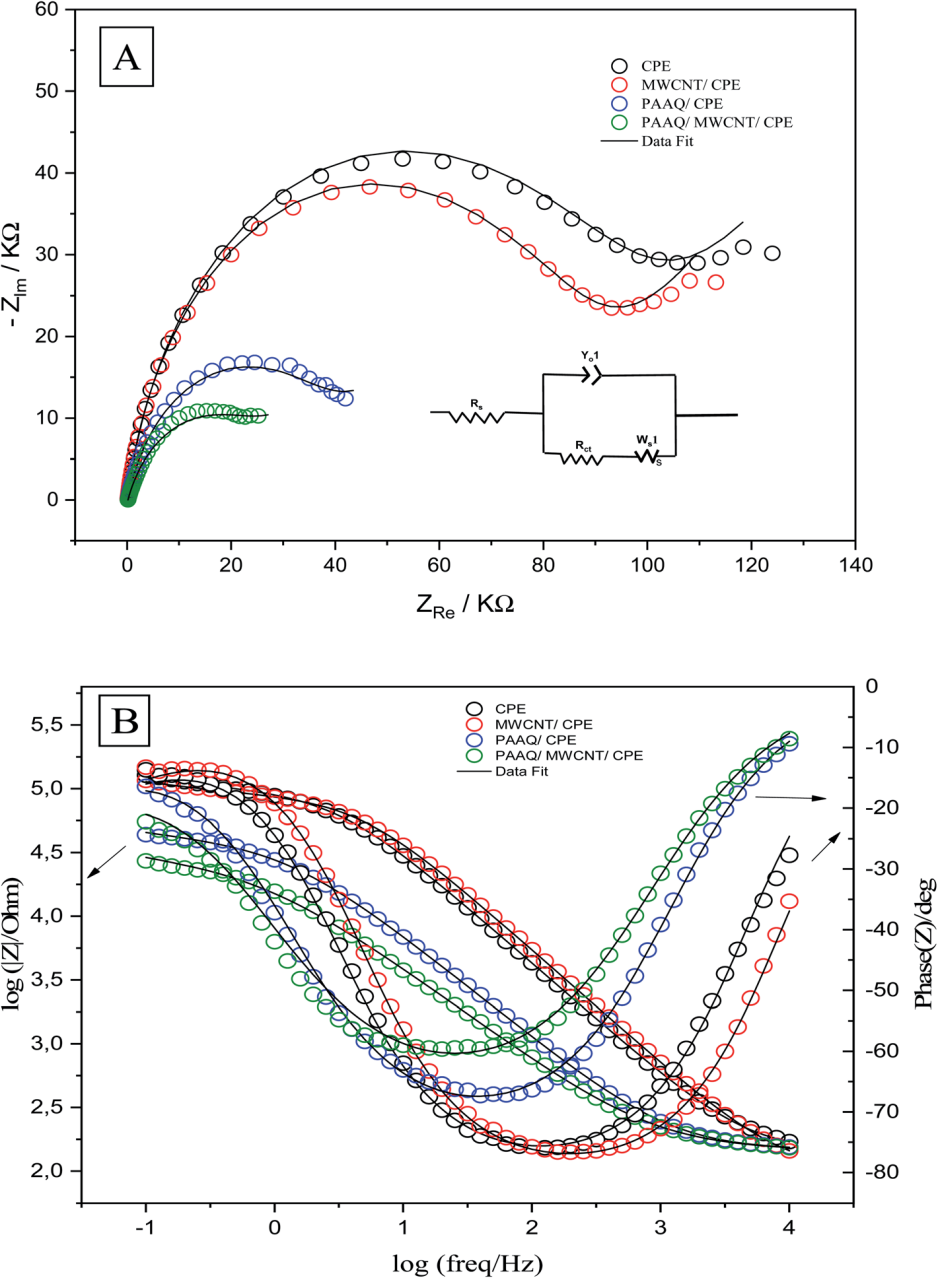
(A) Nyquist and (B) Bode plots of CPE, MWCNT/CPE, p-AAQ/CPE, and p-AAQ/MWCNT/CPE in 0.3 M KCl containing 0.1 M Fe_3_(CN)_6_^3−/4−^—the theoretical fitting data according to the equivalent circuit is shown in the right inset of (A).

The EIS measurements were conducted to determine the charge-transfer resistance (*R*_ct_) at the interface of the different composites. The Nyquist and Bode plots of CPE, MWCNTs/CPE, p-AAQ/CPE, and p-AAQ/MWCNTs/CPE in 0.3 M KCl containing 0.1 M Fe_3_(CN)_6_^3−/4−^ are shown in Fig. 5A and B. Alternating current was applied in a frequency range of 0.1–10 000 Hz. Regarding the voltage, the obtained electrical outputs were sinusoidal at different phase shifts (phase *Z*). The Randles circuit was utilized to fit the experimental data. Therein, *R*_s_ was the solution resistance, and *R*_ct_ is the charge-transfer resistance. Moreover, *Y*_o_ is the constant phase element, and its corresponding component (*n* = 1) represents the capacitance of the double layer. *W*_s_ is the Warburg impedance that is relative to the diffusion of the analyte from the solution to the surface of the electrode. The most valuable component for comparing the modified layers is *R*_ct_ that was 95.18, 86.03, 39.73, and 27.01 kΩ for CPE, MWCNTs/CPE, p-AAQ/CPE, and p-AAQ/MWCNTs/CPE, respectively. p-AAQ/MWCNTs/CPE achieved the highest electron transfer, affording a good correlation with the results obtained from DPSV in presence of Se^4+^.

### 3.4. Optimization of the analytical conditions

**Fig. 6:**
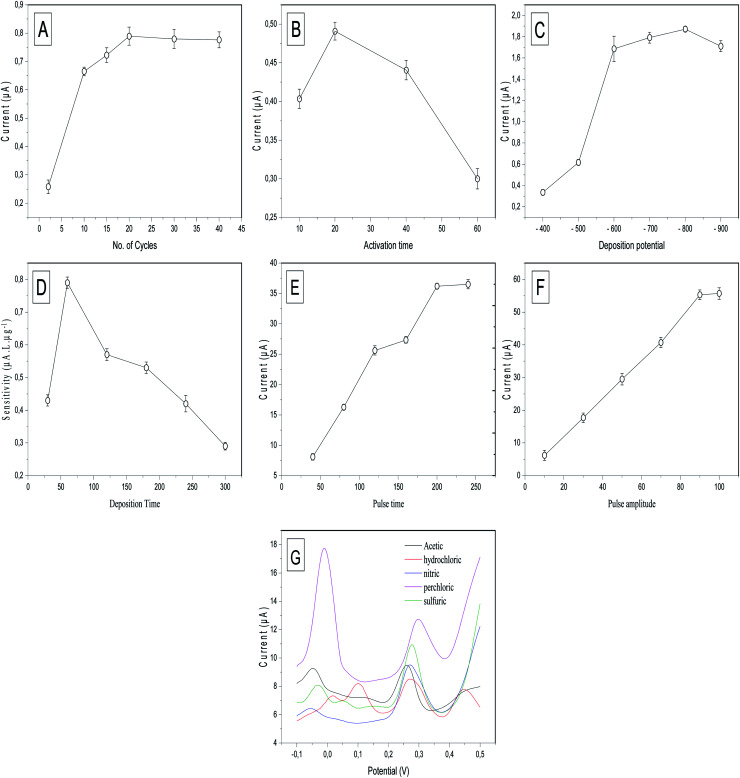
Effect of (A) the number of CV scans in the activation step, (B) activation time, (C) deposition potential from −400 to −900 mV at a stripping peak current of 50 μg L^−1^ Se^4+^ in 0.1 M H_2_SO_4_, (D) effect of the deposition time on the sensitivity of the calibration curve that was drawn between 10, 20, and 40 μg L^−1^ Se^4+^ in 0.1 M H_2_SO_4_; effect of the (E) differential-pulse time in ms and (F) differential-pulse amplitude in mV on the peak current of 50 μg L^−1^ Se^4+^ in 0.1 M H_2_SO_4_, and (G) DPV of 50 μg L^−1^ Se^4+^ in 0.1 M acetic, 0.1 M HCl, 0.1 M nitric acid, 0.1 M perchloric acid, and 0.1 M H_2_SO_4_.

To achieve the maximum sensitivity of the prepared electrode (p-AAQ/MWCNTs/CPE) toward Se^4+^, several experimental conditions, including the p-AAQ formation conditions; the number of scanning cycles and time for the activation; the deposition potential and time; the differential-pulse features, including the pulse time and amplitude; and the electrolyte solution, were studied, and the results were shown in Fig. 6. The results confirmed that the conditions for forming the polymer were pivotal to the determination of Se, and this supported the fact that the detection of Se depended mainly on the presence of p-AAQ. Thickness of the agglomerated polymer on the surface of CPE depended on the number of scanning cycles in the polymerization process, and this was highly related to the amount of Se that was deposited in the preconcentration step, as reflected by the stripping peak current. The scanning cycles were studied from 2 to 40 (Fig. 6A), and a gradual increase in the peak current was observed for 20 cycles. Further, a saturation of the thickness of the polymer was achieved without any further improvement in the peak current. Additionally, the time at which the fixed potential (0.75 V) exhibited a different behavior from that of the number of scanning cycles during the formation of the polymer was investigated from 10 to 60 s (Fig. 6B), and the highest peak current was observed in 20 s. Moreover, as the time increased to ∼40 s, the peak current decreased, and this could be the cause of disordering in the agglomeration.

Furthermore, the effect of the electrodeposition potential at a stripping current of 50 μg L^−1^ Se^4+^ on the proposed sensor was investigated since it is a key parameter in anodic stripping voltammetry. It is defined as the potential at which the metal cations are deposited on the surface of the electrode to commence differential-pulse stripping. The deposition potential was studied from −400 to −900 mV (Fig. 6C). The results indicated that the highest stripping peak current was achieved at −800 mV, and this was attributed to the reduction of Se^4+^ in the form of piaselenol that was assessed using negative potential. By further increasing the negative potential to −900 mV, the peak current decreased owing to the saturation of the piaselenol complex that was formed on the surface of the working electrode.

The deposition time is the period that controlled the number of metal cations that were deposited in the preconcentration step. According to the literature, the deposition time regulates the sensitivity, enhancing the peak current. An increase in the deposition time might negatively affect the sensitivity since sensitivity is defined as the slope of the calibration curve. The results shown in Fig. 6D revealed that 60 s was the optimal deposition time, investigated according to the slope of the calibration curve that was established between 10, 20, and 40 μg L^−1^ Se^4+^. Although the value of the peak current (10 μg L^−1^) increased with increasing deposition time up to 300 s, the sensitivity did not increase because of the saturation of the surface of the working electrode with metal cations of high concentrations at high deposition times.

Differential pulse voltammetry (DPV) is a pulse technique developed to minimise background charging currents. The waveform in DPV is a sequence of pulses in which a baseline potential is held for a time before a potential pulse is applied. The current is measured just before the potential pulse is applied. The potential is then increased by a small amount and the current is measured again at the end of the pulse. The potential of the working electrode is then reset by a smaller value than during the forward pulse, so that the baseline potential is increased at each pulse throughout the sequence.

DPV employs the potential pulses that were produced at a linear potential gradient. DPV combines four features that could be optimized to increase the peak current sensitivity. These features include the potential ramp or step potential (*E*_step_) in mV, height of the potential pulse or pulse amplitude in mV, duration of the pulse potential or pulse width in ms, and time after the pulse or pulse time at which the current can be quantified in ms. The two valuable parameters that were studied to optimize DPV are pulse amplitude and pulse time. Regarding the pulse time, it was studied in the range of 40 to 250 ms (Fig. 6E). By continuously increasing the time up to 200 ms, the peak current did not exhibit any significant increase. Therefore, 200 ms was selected as the optimal pulse time for DPV. Further, the pulse amplitude was varied in the range 10–100 mV, and the differential peak current *versus* the pulse amplitude (Fig. 6F) exhibited a significant increase in the peak current up to 90 mV. However, no increase was observed at 100 mV. Thus, 90 mV was selected as the optimal pulse amplitude for DPV.

Next, the role of the supporting electrolytes is very crucial to the stripping behavior of Se^4+^. The supporting electrolytes contain associated anion species, which affected the charge-carrier transfer. Different supporting electrolytes, such as 0.1 M acetic acid, 0.1 M HCl, 0.1 M nitric acid, 0.1 M perchloric acid, and 0.1 M H_2_SO_4_, were studied for DPSV of 50 μg L^−1^ Se^4+^ (Fig. 6G). Perchloric acid exhibited the highest sensitivity, although it also exhibited the highest background current, which affected the analytical performance of the electrode by increasing the charging current compared with the faradaic current since the perchlorate ions can store the charge at an increased Se^4+^ concentration. Further, H_2_SO_4_ exhibited the highest sensitivity compared with HCl and nitric and acetic acids. Therefore, H_2_SO_4_ was selected as the supporting electrode for DPSV of Se^4+^. Several oxidation peaks occurred in each electrolyte solution, with two distinguishable peaks at −0.01 and 0.31 V in the perchloric acid. The peak at −0.01 is probably due to overlapping peaks of oxidation of adsorbed Se in the bulk and adsorption on the monolayer. This overlap could be due to the adsorption of a monolayer of hydrated perchlorate ions (ClO_4_·2H_2_O).^42^ This also explains the highest background current obtained with perchloric acid. Three oxidation peaks in 0.1 M acetic acid, 0.1 M nitric acid and 0.1 M H_2_SO_4_ at (−0.05, 0.09 and 0.25 V), (−0.05, 0.01 and 0.027 V) and (−0.04, 0.03 and 0.27 V), respectively, are easily explained by the previous eqn (1)–(3). Four oxidation peaks occurred in 0.1 M HCl. The first oxidation peaks at 0.01, 0.07 and 0.3 V can be easily explained by the previous equations, while the fourth peak at 0.45 V can be explained by the formation of the selenium monochloride–Se–Cl bond.^43^

Overall, for the anodic polymerization of 1-AAQ, cyclic voltammetry should be performed for 20 cycles and fixed potential applied for 20 s. Deposition potential of −800 mV should be performed for a deposition time of 60 s for electrodeposition of Se^4+^. Afterward, DPV should be deployed for the stripping step with pulse time of 200 ms and pulse amplitude of 90 mV. H_2_SO_4_ was chosen as the optimal electrolyte medium with minimal background current and high sensitivity obtained for Se^4+^.

### 3.5. Electrochemical impedance spectroscopy of Se^4+^on the studied modified electrodes

**Fig. 7:**
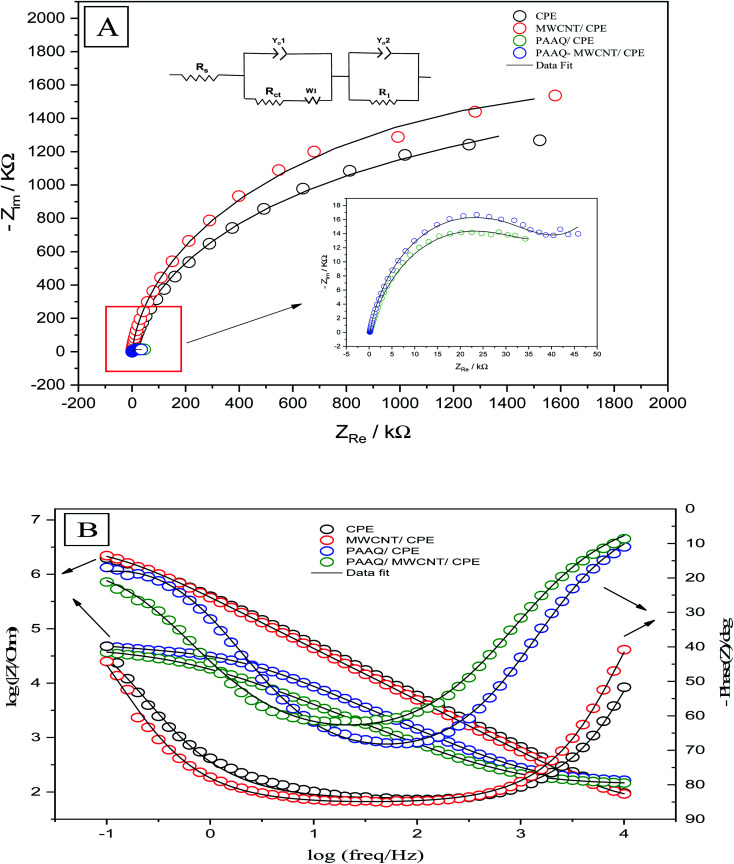
(A) Nyquist and (B) Bode plots of CPE, MWCNT, p-AAQ/CPE, and p-AAQ/MWCNT/CPE in 0.1 M H_2_SO_4_ containing 50 μg L^−1^ Se^4+^—the theoretical fitting data based on the equivalent circuit is shown in the lift inset of (A).

Electrochemical impedance spectroscopy determines the electrochemical properties of the analytical system by measuring the correct positions of the components of the electrochemical circuit (resistors, capacitors, transistors) and their effects on the movement of ions and electrons by applying an alternating current (AC) and measuring their responses as functions of frequency. EIS was performed to obtain information about the surface properties of the working electrodes. The rate of electron transfer between the electrolyte and the electrode surface was determined by EIS. The positions of the components of the electrochemical cell (resistors, capacitors, *etc.*) were evaluated by means of an equivalent circuit which was used to fit the experimental data to the Nyquist (Fig. 7A) and Bode (Fig. 7B) diagrams. EIS was performed in a solution of 0.1 M H_2_SO_4_ containing 50 μg L^−1^ Se^4+^ on CPE, MWCNTs/CPE, p-AAQ/CPE and p-AAQ/MWCNTs/CPE. Randles' modified equivalent circuit was used to fit the experimental data. The two constant phase components are represented by *Y*_o1_ and *Y*_o2_, *R*_1_ represents the impedance caused by the host–guest interaction between p-AAQ and Se^4+^. The values of *R*_ct_ were 1853, 2740, 38.531 and 39.539 kΩ for CPE, MWCNT/CPE, p-AAQ/CPE and p-AAQ/MWCNT/CPE, respectively. The results showed the high affinity and low resistance of the p-AAQ modified electrodes to Se; they also showed the slightly better performance of p-AAQ/MWCNT/CPE than p-AAQ/CPE.

**Fig. 8:**
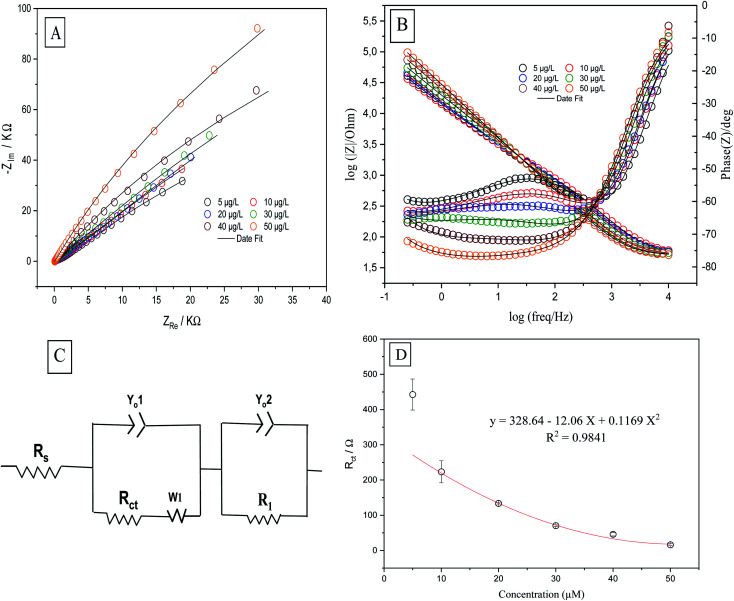
(A) Nyquist impedimetric and (B) Bode plots of 5, 10, 20, 30, 40, and 50 μg L^−1^ Se^4+^ on p-AAQ/MWCNT/CPE in 0.1 M H_2_SO_4_; (C) equivalent circuit for fitting the experimental data; and (D) polynomial calibration curve based on the *R*_ct_ values.

Further, EIS was performed to test p-AAQ/MWCNTs/CPE at different concentrations of Se^4+^ to evaluate the analytical performance of the proposed sensor toward the analyte through the observation of the impedimetric behavior. The Nyquist and Bode plots (Fig. 8A and B) were employed to evaluate the EIS data in which the experimental data were fitted according to the equivalent circuit (Fig. 8C) that was composed of similar components as that of the previous experiment. The polynomial calibration curve that was established between the concentrations of *R*_ct_ and Se^4+^ exhibited a reverse relationship with the increasing concentration of Se. The calibration curve was established in a concentration range of 5–50 μg L^−1^ Se^4+^ (Fig. 8D), obtaining the following regression equation: *y* = 328.4 − 12.06*x* + 0.1169*x*^2^, with an LOD value of 1.5 μg L^−1^ (3*σ*) and a coefficient of detection (*R*^2^) of 0.9841. Moreover, a good *R*^2^ value indicated the good analytical performance of p-AAQ/MWCNTs/CPE toward various concentrations of Se^4+^ in 0.1 M H_2_SO_4_; the same behavior was observed by DPV, as discussed in Subsection 3.6.

### 3.6. Analytical performance

**Fig. 9:**
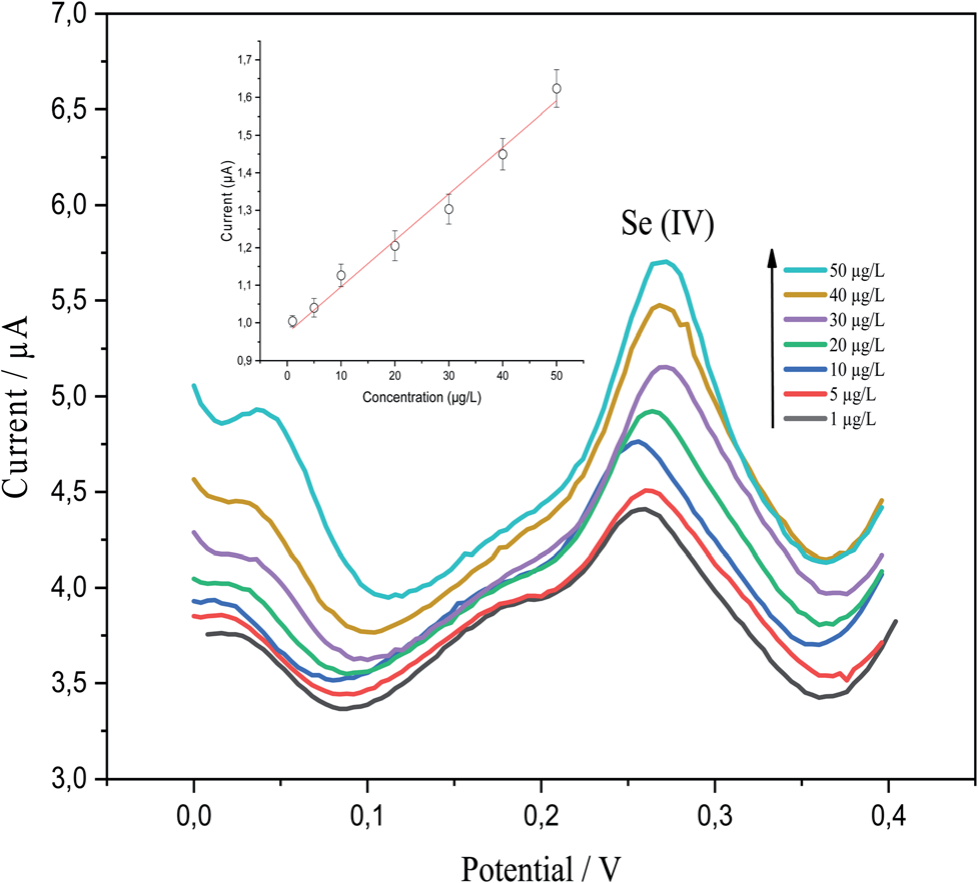
Differential-pulse voltammograms of 1, 5, 10, 20, 30, 40, and 50 μg L^−1^ Se^4+^ in 0.1 M H_2_SO_4_ at a deposition time of 60 s, a deposition potential of −0.8 V, a differential-pulse time of 200 ms, a differential-pulse amplitude of 90 mV, a pulse width of 50 ms, and a step potential 1 mV with the corresponding inset of the calibration curve.

p-AAQ/MWCNTs/CPE demonstrated good analytical performance toward various Se^4+^ concentrations, as revealed by DPV under the preoptimized conditions. This was clearly revealed by the linear behavior between the concentration of Se^4+^ and the stripping peak currents. P-AAQ/MWCNTs/CPE exhibited good peaks of Se^4+^ at 0.27 V with a shift in the potential either to the left or right side probably because of the drop in IR.^44^Fig. 9 shows DPVs of the multipoint standard additions of Se^4+^ in 0.1 M H_2_SO_4_ with the inset of the calibration curve. A linear relationship was obtained in the concentration range of 1–50 μg L^−1^ with LOD and sensitivity of 0.25 μg L^−1^ (3*σ*) and 0.98 μA L μg^−1^, respectively. LOD was determined as mentioned in the Experimental section

5 LOD = 3*σ*,

where *σ* is the standard deviation of 10 measurement of lowest concentration (1 μg L^−1^ Se^4+^).

The linear regression equation is expressed, as follows: *I*_p_ (μA) = 0.98071*C*_Se^4+^_ (μg L^−1^) + 0.0121 (*R*^2^ = 0.9857).

P-AAQ/MWCNT/CPE was demonstrated as a novel, unique, and rapid electrochemical sensor for Se^4+^ compared with other electrochemical Se^4+^ sensors, as listed in Table 1. The proposed sensor obtained a lower LOD value compared with other reported ones.^4,41,45^ Additionally, although several reported modified electrodes obtained lower LOD values than did our proposed sensor, our sensor demonstrated great performance regarding simplicity, robustness, ease of handling, and ease of preparation.

**Table 1:** Comparison of p-AAQ/MWCNTs/CPE with other modified electrodes for the determination of Se^4+^

Modified electrode	Technique	Peak potential (V)	Linear range (μg L^−1^)	LOD (μg L^−1^)	Ref.
Fe(OH)_3_/SMDE	DPCSV	Not given	0.01–0.1	0.02	46
Graphite SPE	ASV	0.8	10–100	4.9	45
DAB-Nafion/MFE/GC	SWCSV	−0.42	0.5–50	0.1	47
AuNPs/GC	SWASV	0.9	1–50 000	0.34	4
Bi/Hg film	AdsDPCSV	−0.55	2–50	0.07	48
Au/ZnO/ITO	CSPE	0.85	5–100	2.89	41
AuNDs/P-rGO	SWASV	0.8	3–300 nM	0.9 nM	49
P-AAQ/MWCNTs/CPE	DPASV	0.27	1–50	0.289	This work

### 3.7. Repeatability of the modified electrode

**Fig. 10:**
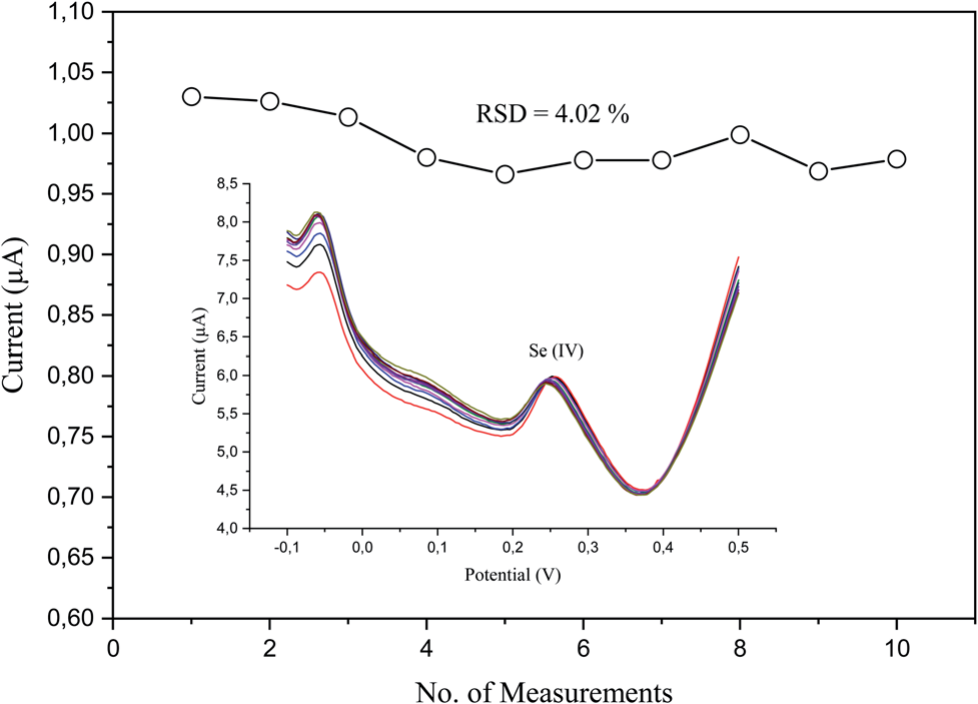
Peak current measurements of 5 μg L^−1^ Se^4+^ on p-AAQ/MWCNTs/CPE in 0.1 M H_2_SO_4_ with the corresponding relative standard deviation with the inset exhibiting the repetitive differential-pulse voltammograms.

The analytical repeatability and stability of our sensor were measured in a standard solution of a low concentration (5 μg L^−1^) of Se^4+^ in 0.1 M H_2_SO_4_ under the preoptimized conditions (Fig. 10). The repeatability was measured regarding the relative standard deviation (RSD), which was 4.02% (*n* = 10). The obtained results revealed its good performance, absence of fouling, and complete oxidation in the stripping step.

### 3.8. Effects of interfering ions

In addition to the evaluation of the sensitivity and repeatability, the selectivity of the modified electrode was tested in the presence of other metal cations. The selectivity test is required before the application of the proposed sensor in real water samples. To evaluate the effects of interfering ions, different cation and anion species, such as Na^+^, Ca^2+^, Mg^2+^, Al^3+^, Cd^2+^, Pb^2+^, Cl^−^, SO_4_^2−^, CO_3_^2−^, and NO_3_^−^ on the 10-, 20-, and 50-fold concentrations were added to a solution containing 2, 5, and 10 μg L^−1^ Se^4+^ under the preoptimized conditions. The effect of the interfering ions on the peak current of Se^4+^ was negligible in cases of 5 and 10 μg L^−1^ Se^4+^ (the signal change was <5%, as confirmed by the tolerance limits).^50^ However, some negative effects were observed in the case of 2 μg L^−1^ Se^4+^ (the peak current was depleted by ∼12%), which could be due to the pretreatment of the samples before the analysis.

### 3.9. Real samples analysis

Wastewater samples were analysed with our sensor to evaluate its practical performance in the analysis of real water samples (Table 2). As mentioned earlier, the presence of other interfering ions does not greatly affect the analytical response (*i.e.* peak current). The regression equation that can be used for the analysis of Se^4+^ in wastewater samples was established for five standard concentrations (2, 5, 10, 30 and 60) prepared in wastewater samples and was calculated as *I*_p_ (μA) = 0.975*C*_Se_ (μg L^−1^) + 0.0098. The sensitivity (*i.e.* the slope of the calibration) is only slightly affected when comparing the curve obtained with deionized water (0.9807) with the calibration curve for standards prepared in waste water (0.975). Two wastewater samples (sample 1 and 2) were spiked with two concentrations of Se^4+^ (10 and 30 μg L^−1^) and the measured concentrations were compared with the assigned concentrations to obtain recovery values. Good recoveries of 95–109% were obtained. The results obtained show that the proposed sensor can be used for the analysis of real water samples.

**Table 2:** Analysis of Se^4+^ in wastewater samples (*n* = 5)

Sample	Added Se^4+^ (μg L^−1^)	Found by p-AAQ/MWCNTs/CPE (μg L^−1^)	Recovery
Waste water sample 1	10	9.49 ± 1.03	95%
	30	32.7 ± 2.6	109%
Waste water sample 2	10	10.87 ± 0.78	109%
	30	28.9 ± 1.8	97%

## 4. Conclusion

In summary, p-AAQ/MWCNTs/CPE was fabricated by an applied potential, followed by CV. SEM, employed to characterize the morphology of our sensor, revealed the images of the proposed candidate. SEM and EDS were also applied to investigate the preconcentration of Se^4+^, and the results indicated the formation of a piaselenol complex on the working electrode surface. The conditions, such as the time of the applied potential and thickness of the polymer film, for preparing the polymer were investigated. A deposition potential of −0.8 V, a deposition time of 60 s, a DPV pulse-time of 200 ms, a DPV pulse-amplitude of 90 mV, and 0.1 M H_2_SO_4_ were selected as the optimal analytical conditions. EIS was applied to investigate the charge-transfer properties of the modified electrode in a hexacyanoferrate solution. Furthermore, the performance of the stripping behavior of Se^4+^ on the various modified electrodes and the variation in the concentrations of Se^4+^ on p-AAQ/MWCNTs/CPE were studied by EIS. The obtained results exhibited good agreement with those, which were obtained by DPV with good linearity (*R*^2^ of 0.986), high sensitivity (LOD of 0289 μg L^−1^ Se^4+^), and good repeatability (RSD = 4.02% for 5 μg L^−1^ Se^4+^). The proposed sensor also exhibited a very good anti-interference behavior toward common ions in wastewater samples. Thus, satisfactory recovery values were obtained after applying the proposed sensor to determine Se^4+^ in two real water samples. Future work will focus on the use of the screen-printed electrode modified by the polymer film (p-AAQ) in a flow injection system for on-site environmental monitoring of Se^4+^ in river water.

## Conflicts of interest

There are no conflicts of interest to declare.

## Supplementary Material

RA-012-D1RA07588H-s001
