# Supplementary material for: Voltammetric and impedimetric determinations of selenium(iv) by an innovative gold-free poly(1-aminoanthraquinone)/multiwall carbon nanotube-modified carbon paste electrode
Source: RSC Adv. 2022 Feb 10;12(8):4988–5000. doi: 10.1039/d1ra07588h (PMC8981389; doi:10.1039/d1ra07588h)
Supplement: RA-012-D1RA07588H-s001 [file RA-012-D1RA07588H-s001.pdf]

Electronic Supplementary Material (ESI) for RSC Advances.
This journal is © The Royal Society of Chemistry 2022

Voltammetric and Impedimetric Determinations of Selenium (IV) by An Innovative Gold-Free Poly(1-aminoanthraquinone)/Multiwall Carbon Nanotubes-Modified Carbon Paste Electrode

Asmaa Galal Ali [a]*, Mahmoud Fatehy Altahan [b,1]**, Amr Mohamed Beltagi [c] Abla Ahmed Hathoot [a], Magdi Abdel-Azzem [a]

[a] Electrochemistry Laboratory, Chemistry Department, Faculty of Science, Menoufia University, Shibin El-Kom 32511, Egypt

[b] Central Laboratory for Environmental Quality Monitoring, National Water Research Center, El-Qanater El- Khairia 13621, Egypt.

[c] Chemistry department, Faculty of Science, Kafrelsheikh University, Kafr El-Sheikh 33516, Egypt.

[1]Current address: GEOMAR, Helmholtz Center for Ocean Research, Kiel 24148, Germany, Email: maltahan@geomar.de.

Corresponding authors email: asmaa.galal081986@gmail.com (Asmaa Galal Ali)

mahmoud_abdalqader@nwrc.gov.eg (Mahmoud Fatehy Altahan)

Supplementary Information

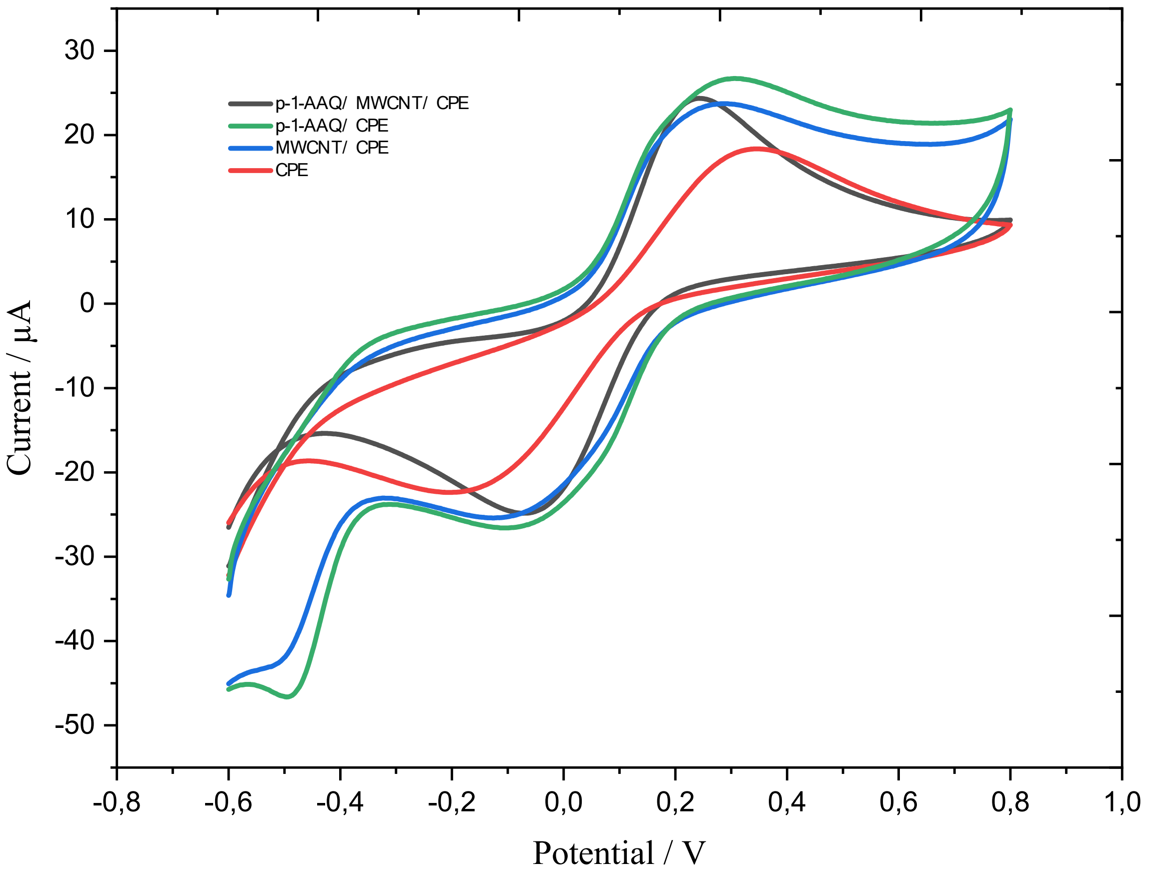

Figure S1. CVs of CPE, MWCNT/CPE, p-1-AAQ/CPE and p-1-AQQ/MWCNT/CPE into 0.001 M $[Fe(CN)_6]^{3-/4-}$ containing 0.1 M KCl at scan rate 0.1 $V.S^{-1}$.

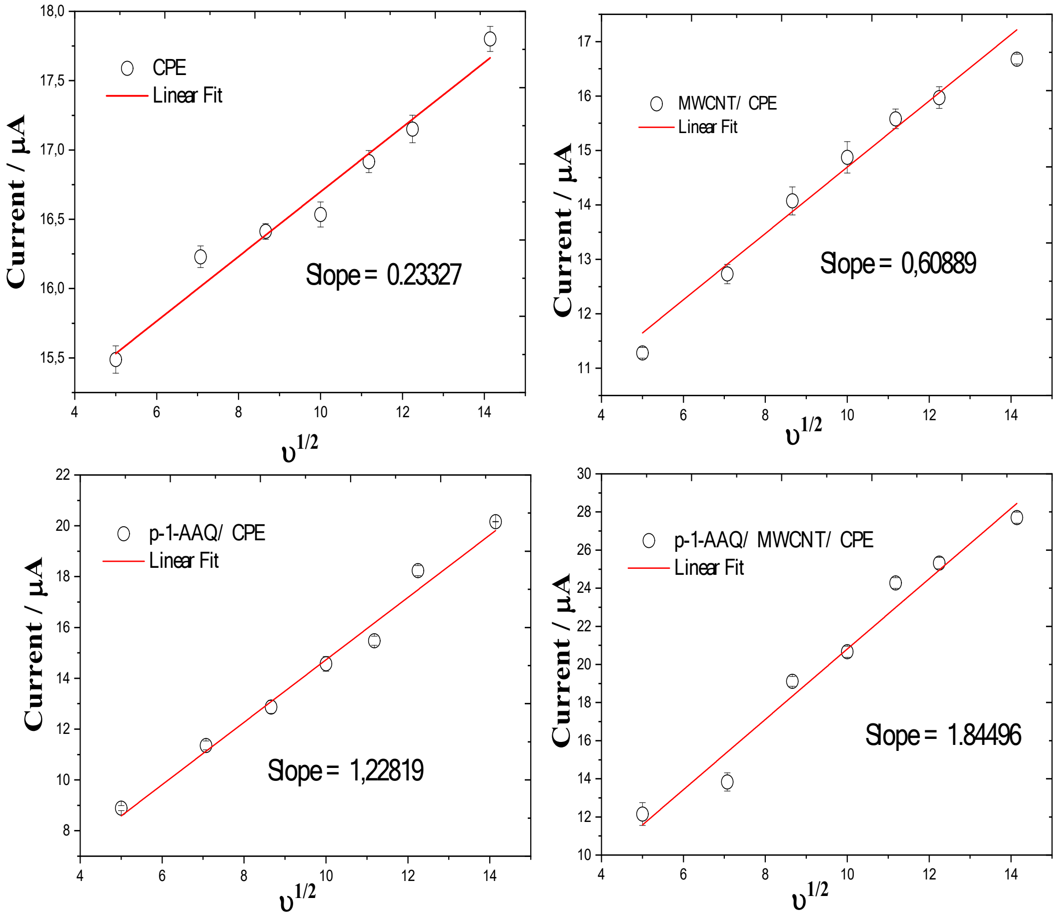

Figure S2. Peaks current Vs. square root of Scan rates curves for CPE, MWCNT/CPE, p-1-AAQ/CPE and p-1-AQQ/MWCNT/CPE into 0.001 M $[Fe(CN)_6]^{3-/4-}$ containing 0.1 M KCl at different scan rates (25 – 200 $mV.S^{-1}$).
